# Circular RNAs: New players involved in the regulation of cognition and cognitive diseases

**DOI:** 10.3389/fnins.2023.1097878

**Published:** 2023-02-02

**Authors:** Xiaohan Yu, Haoyu Liu, Ning Chang, Weijia Fu, Zhiwen Guo, Yue Wang

**Affiliations:** ^1^School of Clinical and Basic Medical Sciences, Shandong Provincial Hospital Affiliated to Shandong First Medical University, Jinan, China; ^2^Medical Science and Technology Innovation Center, Shandong First Medical University and Shandong Academy of Medical Sciences, Jinan, China

**Keywords:** circular RNAs (circRNAs), cognition, aging, Alzheimer’s diseases, depression, memory, autism spectrum disorder (ASD)

## Abstract

Circular RNAs (circRNAs), a type of covalently closed endogenous single-stranded RNA, have been regarded as the byproducts of the aberrant splicing of genes without any biological functions. Recently, with the development of high-throughput sequencing and bioinformatics, thousands of circRNAs and their differential biological functions have been identified. Except for the great advances in identifying circRNA roles in tumor progression, diagnosis, and treatment, accumulated evidence shows that circRNAs are enriched in the brain, especially in the synapse, and dynamically change with the development or aging of organisms. Because of the specific roles of synapses in higher-order cognitive functions, circRNAs may not only participate in cognitive functions in normal physiological conditions but also lead to cognition-related diseases after abnormal regulation of their expression or location. Thus, in this review, we summarized the progress of studies looking at the role of circRNA in cognitive function, as well as their involvement in the occurrence, development, prognosis, and treatment of cognitive-related diseases, including autism, depression, and Alzheimer’s diseases.

## Introduction

Hsu and Coca-Prados (1979) first reported the circular form of RNA in the cytoplasm of eukaryotic HeLa cells by electron microscopy. However, for a long time, circular RNAs (circRNAs) have been regarded as the byproducts of the aberrant splicing of genes without any biological functions (Sanger et al., 1976; Capel et al., 1993; Cocquerelle et al., 1993; Zaphiropoulos, 1996). Recently, with the development of high-throughput sequencing and bioinformatics, thousands of circRNAs have been confirmed in multiple species and were found to be highly conserved with separate regulation from their linear counterparts, thus attracting increasing attention from scientists (Rybak-Wolf et al., 2015; Gokool et al., 2020). Except for the great advances in understanding circRNA roles in tumor progression, diagnosis, and treatment (Meng et al., 2017; Zhang and Xin, 2018; Kristensen et al., 2022), accumulated evidence has shown that circRNAs are abundantly expressed in the brain compared with other tissues (Rybak-Wolf et al., 2015; You et al., 2015; Hanan et al., 2017).

To be more precise, most circRNAs are enriched in the synapse, including the presynaptic active zone, the presynaptic membrane, and postsynaptic density (Rybak-Wolf et al., 2015; Zajaczkowski and Bredy, 2021). Synapses are the key regions where neurons can communicate with each other and further form specific neural networks involved in higher-order cognitive functions. Additionally, circRNAs change dramatically in two phases, namely, brain development and aging, which are closely related to the rise and fall of cognitive function, suggesting that they have the potential to be important regulators of normal cognitive function (Rybak-Wolf et al., 2015; Xu et al., 2018; Mahmoudi and Cairns, 2019). Thus, aberrant expression or location of circRNAs may also be involved in many cognitive-related diseases (Akhter, 2018; Mehta et al., 2020).

In this review, we outline the recent progress in the biogenesis, turnover, properties, and biological roles of circRNAs, especially focusing on what the potential effects of circRNAs are on neural cognitive functions and how abnormal circRNA expression and dysfunction are associated with the pathogenesis of some cognitive-related diseases, including autism spectrum disorder (ASD), major depressive disorder (MDD), and some aging-related diseases, such as Alzheimer’s disease (AD). In addition, the possibility of using circRNAs as clinical diagnostic biomarkers and therapeutic targets in these diseases is also elaborated.

## Biogenesis and turnover of circRNAs

### Biogenesis

CircRNAs can be derived from the back-splicing of canonical splice sites, which is different from the canonical splicing mode of linear RNAs (Salzman et al., 2012; Jeck et al., 2013; Memczak et al., 2013; Zhang et al., 2014). A simple description of back-splicing is that the downstream sequence of a precursor mRNA is inversely connected with its upstream RNA sequence to form the circRNA. Although the complete mechanism of circRNA biogenesis remains unclear, three main possible formation models of circRNAs are fairly well accepted.

#### Direct back-splicing

**FIGURE 1:**
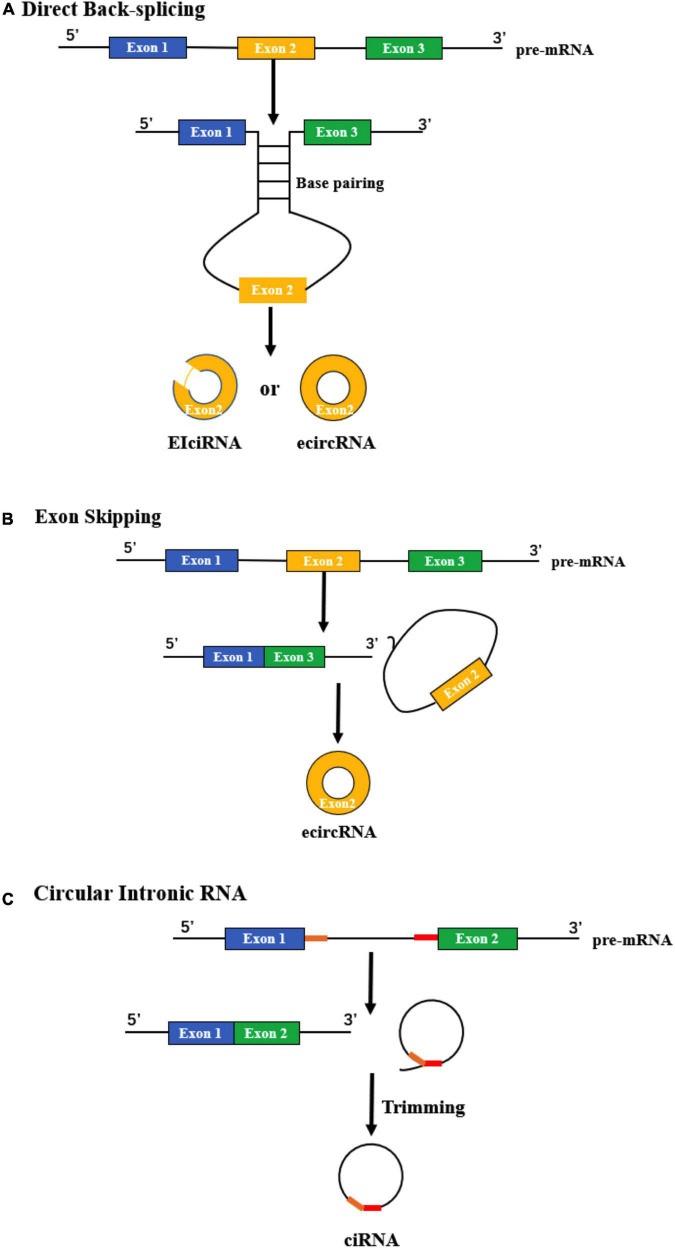
Biogenesis of circRNAs. **(A)** Direct back-splicing occurs when an upstream intron and downstream intron pair with each other through complementarity, forming ecircRNAs or EIciRNAs. **(B)** The process of exon skipping begins with the splicing of pre-mRNA. Then, a lariat containing the exon (yellow) and introns is produced, after which an ecircRNA is generated. **(C)** The formation of ciRNA depends on two significant RNA elements (yellow and red), which enable an intron to escape debranching and degradation.

Research shows that direct back-splicing is the main mechanism of circRNA formation (Pan et al., 2020). During this process, a circular structure is formed by looping the intron sequences flanking the downstream splice-donor site with the upstream splice-acceptor site. Base pairing of two introns via short repeat sequences is one important pattern to bring these sites into close proximity (Kristensen et al., 2019). One report by Liang and Wilusz (2014) found that the Alu family played an important role in the circularization of some exons, and only a small portion of them was sufficient to form circRNAs. Interestingly, they found that longer repeat parts were not necessarily better for circularization; in contrast, some may even play an inhibitory role (Liang and Wilusz, 2014). RNA-binding proteins (RBPs) that bind to specific motifs in the flanking introns can also facilitate the formation of circRNAs during direct back-splicing. For example, Quaking changed during the epithelial–mesenchymal transition could specifically bind with the motif in introns and positively regulate the abundance of circRNAs (Conn et al., 2015). Errichelli et al. (2017) reported that another RBP, FUS, a key protein linked to the pathogenesis of amyotrophic lateral sclerosis and frontotemporal dementia, regulated circRNA biogenesis by binding the introns flanking the back-splicing junctions. After forming the circular structure, some introns are then removed, and exonic circRNAs (ecircRNAs) or exon-intron circRNAs (EIciRNAs) are generated (Figure 1A).

#### Exon skipping

During this process, an exon “skips” from the final mRNA product, and sequences between its upper and downstream exons can form an exon-intron lariat structure. Then, the lariat undergoes internal splicing and finally generates a circRNA (Petkovic and Muller, 2015) (Figure 1B). This phenomenon is common in lower eukaryotes. For example, in a type of yeast named *Schizosaccharomyces pombe*, *mrps16* gene lacks any base pairing between the regions flanking the circularized exon, which means that the method to form *mrps16* circRNA is quite likely to be different from direct back-splicing. Through the quantitative analysis of isoforms, it has been demonstrated that exon skipping is the main mechanism of circRNA biogenesis in *mrps16* (Barrett et al., 2015). Kelly et al. (2015) also verified exon skipping in primary human endothelial cells. They used a custom pipeline to characterize circRNAs derived from coding exons and found that circularization of exons is widespread and correlates with exon skipping (Kelly et al., 2015).

#### Formation of circular intronic RNAs (ciRNAs)

CiRNA can be derived from an intron lariat, which is quite different from back-splicing and exon skipping. An intron lariat is produced due to the failure of debranching, and the formation of a ciRNA is related to specific consensus motifs, which contain key RNA elements near the 5’ splice site and the branchpoint (Figure 1C). These critical elements can produce a marked effect through the promotion of inefficient debranching, making it possible to generate a ciRNA (Zhang et al., 2013).

### Turnover

Due to the special circular structure of circRNAs, they are unlikely to become the primary targets of exonucleases or some exogenous chemicals, which enables them to have higher stability than their parental linear RNAs (Zhang L. et al., 2020). Due to this structure, and except for some methods similar to linear RNAs, circRNAs also have some unique ways for their turnover.

Currently, there are more than 160 chemical modifications identified for RNAs, and methylation is the most common type of these modifications (Boccaletto et al., 2022). Of all the types of methylation modifications, *N*^6^-methyladenosine (m^6^A) modification is the most frequently found type in eukaryotic cells (Wang et al., 2020c). The m^6^A modification plays a significant role in the regulation of RNA, including turnover. One previous study reported that m^6^A-modified circRNAs can be cleaved through the endoribonucleolytic cleavage pathway with the help of HRSP12, YTHDF2, and RNase P/MRP (Park et al., 2019). HRSP12 is one kind of adapter and is responsible for bridging YTHDF2 and RNase P/MRP to form a complex. Once the mA-modified circRNA is distinguished by YTHDF2, it can be cleaved by RNase P/MRP and downregulated as a result, which means that m^6^A modification is a way to degrade circRNAs.

Another study showed that the decay of circRNAs can be initiated with the binding of miRNA, which appears to be a distinctive method for cerebellar degeneration-related protein 1 antisense transcript (circRNA CDR1as) (Hansen et al., 2011). There is a miR-671 target site on CDR1as, which makes it possible for CDR1as to be cleaved by Argonaute 2 (Ago2). In addition, circRNAs can also be transferred between cells through exosomes. CiRS-122 is reported to be delivered from drug-resistant cells to drug-sensitive cells with the help of exosomes, which indicates that the decreases in circRNAs in drug-resistant cells may mainly occur through blocking the release of exosomes (Wang et al., 2020d).

## Categories and properties of circRNAs

### Categories of circRNAs

According to the composition of different circRNAs, they can be classified into three types: ecircRNAs, EIciRNAs, and ciRNAs (Zhang et al., 2013; Li et al., 2015; Petkovic and Muller, 2015). The most common type identified in studies is ecircRNAs, which are mainly located in the cytoplasm, while the other two types are predominantly located in the nucleus (Li et al., 2015; Pan et al., 2020). Because of the different cellular localizations, their biological functions and the way they act are completely distinct (Chen, 2020).

### The properties of circRNAs

Based on current research, some notable characteristics of circRNAs have been identified. First, circRNAs have better stability than linear RNAs. Due to the closed loop structure, which lacks 5′ caps and 3′ poly(A) tails, circRNAs are quite stable and can resist the degradation of RNAase R (Qu et al., 2015). Second, circRNAs have a wide variety and distribution. It has been shown that circRNAs widely exist in the biological world and can be detected in many species, from viruses to plants and animals. In addition, circRNAs account for 5–10% of the parent gene transcripts and are a major transcript with higher abundance than mRNAs (Danan et al., 2012; Jeck et al., 2013; Guo et al., 2014). Finally, circRNAs can be expressed with tissue/developmental stage specificity, indicating that diverse functions might exist both in physiological and pathological conditions in almost all organs (Rybak-Wolf et al., 2015; Szabo et al., 2015).

## Putative biological functions of circRNAs

### MiRNA sponges

CircRNAs can interact with many molecules, of which miRNAs are important (Ma et al., 2020). There are some miRNA binding sites on circRNAs, similar to the relationship between mRNA and miRNA. One circRNA can bind to many different miRNAs, and a miRNA can also link with multiple circRNAs. CircRNAs are able to competitively bind miRNAs and act as sponges to inhibit the interaction between miRNAs and their target genes. CiRS-7, which harbors more than 70 binding sites for miR-7, is one of the most studied circRNAs related to the development of the brain. As ciRS-7 and miR-7 are both highly expressed in the brain and there are many binding sites on ciRS-7, ciRS-7 can absorb the complexes of miR-7 and Ago2 and subsequently make miR-7 ineffective, which increases the expression level of miR-7 target genes (Hansen et al., 2013; Memczak et al., 2013).

### Interacting with RBPs

Similar to the relationship between circRNAs and miRNAs, circRNAs have also been reported to function as RBP sponges and indirectly regulate downstream signaling pathways (Ashwal-Fluss et al., 2014). For example, circFoxo3, produced by the tumor suppressor gene Foxo3, can play a role in regulating cell cycle progression by interacting with cell division protein kinase 2 and cyclin-dependent kinase inhibitor 1 (Diallo et al., 2019). In addition, circRNA FECR1 can recruit TET1 to the promoter part of its host gene, Friend leukemia virus integration 1, which ultimately induces transcription (Chen N. et al., 2018).

### Acting in protein translation

Since circRNAs are one kind of ncRNA, they are normally considered to be unable to encode proteins, which has been proven to be incorrect now. Although there are no essential elements for cap-dependent translation, such as the 5′ cap and the poly(A) tail, circRNAs can be translated in a cap-independent manner (Li et al., 2018). This special translation may occur with the help of internal ribosome entry sites or m^6^A motifs (Li et al., 2018). One study reported that circFBXW7 can encode the protein FBXW7-185aa, which may have an effect on arresting the cell cycle and reducing the proliferation of glioma cells (Yang et al., 2018). Interestingly, FBXW7-185aa exhibits a different function compared with the protein FBXW7α. FBXW7α can inhibit c-Myc with a ubiquitination-induced degradation manner, and play a tumor-suppressive role (Shen et al., 2022). Unlike FBXW7α, FBXW7-185aa can interact with USP28, a deubiquitinating enzyme that can stabilize c-Myc, and unoccupied FBXW7α to degrade c-Myc.

### Regulation of parental gene transcription

Studies have shown that circRNAs containing introns, such as ciRNAs and EIciRNAs, are mostly distributed in the nucleus and can participate in the expression of their host genes. For example, ci-ankrd52 and ci-sirt7 can take part in the process of modulating parental gene transcriptional activity in *cis* by interacting with RNA polymerase II (Zhang et al., 2013).

## Online databases for circRNAs study

Based on the development of circRNAs sequencing and the in-depth understanding of circRNAs, many online databases with numerous bioinformatic analysis tools have been established, which can facilitate the study of circRNAs from different perspectives. Previous reviews have made a good summary of these databases, including the advantages or disadvantages of them, detailed analysis functions, and applications (Aghaee-Bakhtiari, 2018; Liu et al., 2022a). Due to the limited space, it will not be repeated here. But one thing needs to be noted that these databases are built with different theories and modeling methods, therefore, the results of the same prediction may be inconsistent in different databases, and further experiments are essential.

## Involvement of circRNAs in regulating cognitive function

### Differential expression of circRNAs during brain development and aging

During evolution, the number and proportion of non-coding RNAs in the genome surged with the increase in complexity of organisms, while the number of protein-coding genes remained roughly constant (Zajaczkowski and Bredy, 2021), suggesting that the appearance of higher-order cognitive ability may be related to the increase of regulatory functions of non-coding RNAs, but not to the mRNAs. It has been proven that circRNAs are not only highly abundant in the mammalian brain but also expressed dynamically with the development of organisms (Rybak-Wolf et al., 2015). The evidence acquired from Drosophila, mice, pigs, and humans all showed that circulating circRNAs explode at specific developmental periods in brains, independent of their linear isoform dynamics (Westholm et al., 2014; Rybak-Wolf et al., 2015; Venø et al., 2015). It was shown that 58% of brain circRNAs are developmentally regulated, while only 2% of linear transcripts show this trend (Chen J. et al., 2018). Moreover, the increased circRNAs were largely associated with some key events during neurodevelopment, including axon guidance, dendrite morphogenesis, and neuron differentiation (Chen et al., 2019). This specific expression pattern of circRNAs indicates their important regulatory functions in the development and differentiation of the nervous system. In addition to the developmental stages of the brain, investigators are also interested in the expression of circRNAs in aging brains. After reaching peaks in the late stage of development, most of the circRNAs gradually decreased and finally reached a relatively stable level (Venø et al., 2015). During aging, as brain structure integrity and function decline, many neural circRNAs increase again and accumulate but the mRNA expression of their parental genes decreases (Westholm et al., 2014; Gruner et al., 2016; Xu et al., 2018; Mahmoudi and Cairns, 2019). Surprisingly, many circRNAs also exhibited sex dimorphism in the brain and were observed to target mRNAs associated with aging (Paudel et al., 2020). Given the strong correlation between brain development or aging and cognitive functions such as learning and memory, the differentially changed circRNAs in these two stages might be involved in the regulation of such cognitive functions.

### Distribution of circRNAs in the brain

Tissue distribution specificity is one of the important properties of circRNAs. Recently, reports have shown that circRNAs are highly enriched in mammalian brain tissue, and some of them cannot be detected in other tissues (Venø et al., 2015; You et al., 2015; Mahmoudi and Cairns, 2019). The reasons for the above phenomenon might be that approximately 20% of protein-coding genes can produce circRNAs in the mammalian brain, and in which many of the circRNA-hosting genes are expressed only. Even if those genes are not expressed specifically in the brain, more circRNAs are produced in the brain than in other tissues because there are more Alu elements and relatively long introns in neural genes, which are more likely to circularize (Jeck and Sharpless, 2014; You et al., 2015; Xu et al., 2021).

The brain is anatomically and functionally divided into distinct regions consisting of diverse cell types with complex transcriptome architectures. It has also been proven that the enriched circRNAs vary in different brain areas. A comparison of circRNA expression in different brain regions of mice indicated that circRNAs were mostly enriched in the forebrain, in which the prefrontal cortex had higher expression of circRNAs than the hippocampus (Rybak-Wolf et al., 2015). In addition, the cerebellum is another area with an abundance of circRNA enrichment (You et al., 2015).

CircRNAs existing in distinct types of neural cells have also been studied to clarify the underlying mechanism of them in brain’s functions and diseases. So far, the number of reported circRNAs in neurons is still the largest of all neural cells. Certainly, the expression of circRNAs was also identified in other two important neuroglial cells, astrocytes and microglia. Astrocytic circNF1-419, circHIPK2, circSTAG1, and circHECTD1 have been identified to promote astrocyte activation by regulating targeted proteins or miRNAs (Nagy et al., 2015; Huang et al., 2017; Han et al., 2018). CircDYM is mainly located in microglia and is involved in depression disorder by regulating the activation of microglia (Zhang Y. et al., 2020). In addition to these adult neural cells, there are some circRNAs in neural stem cells (NSCs), such as circSLC45A4, circHIPK2, and cZNF292, which are strongly linked to the proliferation, differentiation, and expansion of NSCs (Wang et al., 2020a).

Subsequently, the exact subcellular localization of circRNAs in brain cells was further studied (You et al., 2015). Gene Ontology (GO) analysis predicts that circRNAs in the brain are mostly derived from genes that are enriched in cellular components such as the presynaptic active zone, presynaptic membrane, synapse, and postsynaptic density. To further confirm this hypothesis, high-resolution *in situ* hybridization (ISH) was used, and it showed that circRNAs are mainly located in both the cell body and the dendrites of neurons (You et al., 2015). These findings suggest that circRNAs are enriched in synapses and may play a role in synaptic plasticity-related cognitive function.

CircRNAs, which can be packaged into extracellular vesicles and exchanged between cells (Cheng et al., 2020; Kuo et al., 2021; Liu et al., 2022b; Pan et al., 2022), are suitable for regulating cognitive function. The execution of cognitive function generally requires the activation and coordination of a large number of related neurons in different regions. The release of circRNAs from the donated cell’s synapse via extracellular vesicles could quickly influence neighboring cells to fine-tune the related neural networks involved in cognition. To date, there is still limited understanding of how these vesicles carrying circRNAs are specifically taken in by effector cells.

#### Effects of circRNA on synaptic plasticity and memory

Previous GO and Kyoto Encyclopedia of Genes and Genomes (KEGG) analyses of circRNAs in the brain revealed that many of them were closely related to the regulation of synaptic plasticity (Bu et al., 2019; Mahmoudi et al., 2019; Filippenkov Ivan et al., 2021), which was supported by some *in vitro* and *in vivo* experiments. For example, a circRNA called circHomer 1 derived from the precursor RNA of Homer scaffold protein 1 is thought to modulate the expression of many genes involved in synaptic plasticity and psychiatric disease (Zimmerman et al., 2020). circGRIA1, a conserved circRNA isoform derived from the AMPA receptor subunit Gria1, shows an age-related and male-specific increase in the prefrontal cortex and hippocampus of rhesus macaques. Knockdown of circGRIA1 in hippocampal neurons promotes synaptogenesis and attenuates age-induced decreases in spontaneous mEPSCs both in amplitude and frequency (Xu et al., 2020). Wang et al. (2020a) showed that circHIPK2 silencing enhanced NSC-differentiated neurons and promoted neuronal plasticity *in vitro* by changing the formation of synapses, thereby helping ischemic brain tissue recover more quickly after stroke. Additionally, Nrf2, a neuroprotective agent, can positively regulate circ-Vps41 expression to increase the number of synaptic vesicles and dendritic spines in the hippocampus and improve impaired memory (Zhang et al., 2022). All of these studies suggest that circRNAs are involved in neuronal plasticity regulation. Our lab, using a fear conditioning model, first screened for the circRNAs that play a role in memory formation, extinction and erasure through the RNA-seq method. We also constructed circRNA-miRNA-mRNA networks in different memory stages according to the competing endogenous RNAs (ceRNA) theory. Through these analyses, we further proved that circRNAs play a role in cognition under normal physiological conditions, and the circRNAs involved in different memory processes vary greatly (Wang, 2021). In the following, more works need to be done to clarify the key circRNAs involved in the specific processes of memory, including fear memory and spatial memory, and their potential mechanisms.

## Involvement of circRNAs in cognitive-related diseases

### CircRNAs and ASD

As a neurodevelopmental disorder, ASD usually occurs before age 3 and lasts for a lifetime, and is characterized by stereotypical interests/behaviors and deficits in social interaction and communication (Lai et al., 2014). The occurrence of ASD is related to abnormal synapse and neuron structure (Varghese et al., 2017). Abnormal enlargement and hyperplasia of the cerebrum (Sparks et al., 2002), volume and neuron number changes in the amygdala and hippocampus (Schumann et al., 2004), and abnormal connections of neurons in different brain regions (Herbert et al., 2003; Just et al., 2007) were found in autistic patients. It has been reported that there are widespread gene expression disorders in autism (Parikshak et al., 2016; Turner et al., 2017), and epigenetic factors are also linked to autism (Sun et al., 2016; Vogel Ciernia and LaSalle, 2016; Wu et al., 2016; Turner et al., 2017). Because of the dynamic expression during neurogenesis, synaptogenesis, and neuronal differentiation (Izuogu et al., 2018), as well as their important roles in epigenetic control over gene expression (Parikshak et al., 2016), circRNA may be involved in the pathogenesis of ASD.

#### Differential expression of circRNAs in ASD

The fact that circRNAs are the most resistant of the RNA species to degradation, they are ideal for postmortem studies. The expression profiles of most circRNAs in autism have been elucidated. Chen et al. (2020) measured circRNA expression levels in the postmortem cerebral cortex of ASD and non-ASD controls and identified 60 circRNAs (22 upregulated and 38 downregulated) involved in ASD. Putative targets of circRNA-microRNA-mRNA axes were enriched in ASD risk genes and genes encoding inhibitory postsynaptic density proteins. By comparing the expression of circRNA in the brains of autistic patients and mice, it was found that the expression of circRNA is highly conserved between humans and mice. In addition, Gasparini et al. (2020) studied the expression profile of circRNA in the hippocampus of BTBR mice, a fully characterized ASD model, and age-matched C57BL/6J mice. In this study, 29 circRNAs were significantly downregulated and 12 were upregulated in BTBR mice. Using another ASD mouse model induced by valproate acid, 1,059 differentially expressed circRNAs (477 upregulated and 582 downregulated) were screened by RNA sequencing. Module analysis further showed that these dysregulated circRNAs correlated with autism-related pathways, including the TGF-beta signaling pathway, Notch signaling pathway, and long-term depression (Wang et al., 2020b).

Regarding the underlying mechanism of these candidate circRNAs in ASD, the ceRNA pattern was proposed in these studies. *In vitro* experiments confirmed that upregulation of circARID1A could increase some ASD risk genes, such as SFARI or AutismKB, by directly sponging miR-204-3p (Chen et al., 2020).

At present, there are relatively few experimental studies on the effect of circRNA on ASD, and most of them are descriptive studies. More experimental and clinical correlation studies need to be done in the future. Therefore, there is still a long way to go for ASD diagnosis and treatment using circRNA.

### circRNA and MDD

Major depressive disorder, characterized by emotional dysfunction, is one of the most familiar mental obstacles in the world. It seriously affects people’s thoughts, feelings, and behaviors and can even lead to suicide. The pathogenesis of MDD is complicated and involves a series of factors, including genetics, neuroendocrinology, epigenetic regulation, and social-psychological factors (Gan et al., 2021). The imbalance of the neural-endocrine-immune regulatory network makes the abnormal secretion of neurotransmitters, hormone, and cytokines, which are closely related to the occurrence of depression (Gold and Chrousos, 2002; Franklin et al., 2012; Lang and Borgwardt, 2013; Yrondi et al., 2018).

#### The differential expression of circRNAs in depression

Given the multiple roles of circRNAs in the regulation of neurotransmitters and synaptic plasticity, the possible involvement of circRNAs in depression has received more attention. Studies have shown that compared with normal subjects, there are changes in the expression of circRNAs in patients with depression, which may be related to the formation mechanism of depression.

Cui et al. (2016) analyzed the expression profiles of circRNAs in peripheral blood mononuclear cells (PBMCs) of patients with depression and healthy controls and identified four differentially expressed circRNAs: hsa_circRNA_002143, hsa_circRNA_103636, hsa_circRNA_100679, and hsa_circRNA_104953. After antidepressant treatment, it was found that only the downregulated hsa_circRNA_103636 had obvious changes, suggesting that hsa_circRNA_103636 may have potential diagnostic value for depression (Cui et al., 2016). Jiang et al. (2017) analyzed by microarray the plasma samples of 7 patients with type 2 diabetes mellitus (T2DM) and 7 patients with T2DM and depression and identified 183 circRNAs that were upregulated and 64 circRNAs that were downregulated. Zhang et al. (2018) comprehensively analyzed the circRNA spectrum of chronic unpredictable mild stress (CUMS) mice with typical depressive symptoms and found that the expression levels of mmu_circ_0001355 and 0001788 were slightly lowered, while the expression of mmu_circ_0001223 was greatly inhibited in the ventral medial prefrontal cortex (vmPFC) of CUMS mice. In addition, they also confirmed that total saponins from the leaves of Panax notoginseng saponins (PNS) can inhibit the development of depression, and deep sequencing shows that a large number of circRNAs are differentially expressed in the vmPFC and hippocampus of CUMS mice treated with PNS (Zhang et al., 2018). Ketamine has an obvious antidepressant effect (Berman et al., 2000). Mao et al. (2020) found five differentially expressed circRNAs in the rat hippocampus after ketamine injection and verified the upregulated rno_circRNA_014900 and downregulated rno_circRNA_005442 by qRT-PCR. Maternal separation (MS) is a stable and reliable method to simulate depression-like behavior. Zheng et al. (2019) examined the whole genome transcriptome of the prefrontal cortex in male rats with MS and found that 20 circRNAs were significantly overexpressed and 17 circRNAs were significantly underexpressed.

#### The role of circRNAs as miRNA sponges in depression

The effects of circRNAs on depression by miRNA sponging are the most studied. As an important posttranscriptional regulator, miRNAs decrease the expression of target genes by binding to their 3′-UTR, which could indirectly be regulated by circRNAs. By using the bioinformatics program RNA hybrid, Huang et al. (2017) found that circHIPK2 contained a target site of miRNA-124. Double ISH showed that circHIPK2 and miRNA-124 colocalize in primary mouse astrocytes, and the expression of miRNA-124 and its downstream target gene sigma non-opioid intracellular receptor 1 are both regulated by circHIPK2 in a ceRNA-dependent manner, thus subsequently affecting the activation of astrocytes (Huang et al., 2017). Due to the important roles of activated astrocytes in depression, circHIPK2 is believed to participate in the formation and development of depression. Zhang et al. (2019) found that the expression level of circHIPK2 in chronic unpredictable stress (CUS) mice was significantly higher than that in the control group. Fecal microbiota transplantation from NLRP3 KO mice significantly alleviated astrocyte dysfunction in CUS-treated recipient mice via inhibition of circHIPK2 expression (Zhang et al., 2019). In addition to discovering the role of circRNAs in astrocytes, studies confirmed another circRNA, circDYM, which is involved in MDD pathogenesis and treatment by sponging miR-9 and regulating the activation of microglia (Zhang Y. et al., 2020). Moreover, circDYM delivered by extracellular vesicles (RVG-circDYM-EVs) significantly increased the expression of circDYM in the brain, inhibited microglial activation, and thus alleviated CUS-induced depression-like behavior (Yu et al., 2022). RVG-circDYM-Evs can easily overcome the obstacles of the blood–brain barrier but also enhance its integrity and inhibit the infiltration of peripheral immune cells (including lymphocytes and myeloid cells) into the brain of CUS mice. This indicates that extracellular vesicle-mediated circDYM delivery is expected to be applied in clinical depression treatment. In addition, Fan et al. (2022) found that the expression of circANKS1B in the dentate gyrus area of CUS rats decreased significantly. This change could decrease the sponging of miR-146a-5p, a microRNA derived from microglial cells, and its downstream target gene KLF4, thus participating in the occurrence of depressive-like behaviors (Fan et al., 2022). These discoveries provide novel insights regarding the specific contribution of circRNAs in the pathogenesis and treatment of depression.

#### The role of circRNAs in depression through the interaction with RBPs

Increasing evidence suggests that the RNA demethylase ALKBH5 is related to MDD (Du et al., 2015). Huang et al. (2020) found that the expression of circSTAG1 in the hippocampus of CUS mice decreased, and the expression of circSTAG1 in the plasma and whole blood of patients with depression decreased significantly. Furthermore, it was found that circSTAG1 had a stronger affinity for ALKBH5 than STAG1 mRNA, especially in astrocytes. When the expression of circSTAG1 was downregulated, ALKBH5 could be released, which reduced the methylation of FAAH and then enhanced the stability of FAAH mRNA, resulting in the dysfunction of astrocytes and the occurrence of depressive-like behaviors (Huang et al., 2020).

#### Perspectives of circRNAs in the diagnosis and treatment of depression

Emerging studies have shown that circRNAs may be involved in the onset and progression of depression, so they have become a promising new field in the diagnosis of depression. Reports have shown that hsa_circRNA_103636 (Cui et al., 2016), hsa_circ_0126218 (Bu et al., 2021), circFKBP8 (Shi et al., 2021), circMBNL1 (Shi et al., 2021), and circDYM (Song et al., 2020) might be promising MDD biomarkers, which may further improve the early detection, effective diagnosis, and convenient monitoring of MDD. However, at present, the diagnostic accuracy and specificity of these circRNAs still need to be verified by multicenter and large samples. Similarly, both *in vivo* and *in vitro* experiments indicated that some circRNAs, including circHIPK2 (Zhang et al., 2019), circDYM (Zhang Y. et al., 2020; Yu et al., 2022), circSTAG1 (Huang et al., 2020), and mmu_circ_0001223 (Zhang et al., 2018) might be drug targets for depression treatment, although there are still few effective anti-depression drugs targeting these targets. Recently, the transportation of exogenous circRNAs or their interfering RNAs into the brain by extracellular vesicles has achieved the same effects as local brain region microinjection on cognitive-related diseases (Yu et al., 2022). Since it is a much easier way to change the expression of circRNAs in the brain, it is expected to be used in clinical practice in the future. However, before this clinical application, more research is needed, especially regarding the safety, efficacy, and cost of circRNA delivery systems.

#### CircRNAs and aging-related cognitive disorders

Aging is a degenerative change of the body due to the increase in age. In the nervous system, it is mainly reflected in the structural degeneration and the decline of physiological functions, such as cognitive impairment and reduced stress response ability, which lead to the occurrence of age-related diseases (Cai et al., 2019). Studies have shown that circRNAs seem to accumulate specifically in the brains of various aging organisms, including Drosophila, mice, and humans (Knupp and Miura, 2018; Xu et al., 2021) and are involved in cell aging and survival, thus regulating aging-related disorders (Cai et al., 2019). However, the mechanisms accounting for the increased levels of circRNAs during aging are still unclear. A special ring structure resistant to degradation by exoribonucleases, the postmitotic nature of neurons, global changes in splicing patterns, and all possible circRNA turnover regulators during aging in the brain are mostly involved in the increased levels of circRNAs (Jeck et al., 2013; Deng et al., 2014; Enuka et al., 2016; Errichelli et al., 2017; Knupp and Miura, 2018).

The high stability and specificity of circRNA expression make circRNA a potential biomarker of human disease and one of the methods for disease diagnosis and prognosis. In particular, some circRNAs accumulated rapidly during brain aging compared with their corresponding mRNAs, suggesting that circRNAs may be sensitive biomarkers for aging. For example, in the analysis of human peripheral blood, the levels of hsa_circ_1305 and hsa_circ_722 in the elderly are higher than those in the young (Dluzen et al., 2018). CircDEF6, circFOXO3, circEP300, and circFNDC3B are confirmed to be related to the human aging phenotype (Haque et al., 2020). These screened circRNAs are helpful for determining the physical condition and initiating early prevention of some aging-related diseases, such as Alzheimer’s disease and Parkinson’s disease. Due to length limitations, this review will mainly focus on the relationship between circRNAs and AD.

#### CircRNAs as biomarkers and therapeutic targets in AD

AD is a neurodegenerative disease with insidious onset and slow progression and is also the leading cause of cognitive dysfunction in the aging population. To date, the neuropathological features of AD are thought to involve excessive amyloid β (Aβ) aggravation, hyperphosphorylation of Tau-induced neurofibrillary tangles (NFTs), and neuronal damage (Jeong, 2017; Ma et al., 2017; Reiss et al., 2018; Naseri et al., 2019). Excessive Aβ causes neuronal inflammation, oxidative stress, and Ca^2+^ overload, which may lead to neuronal damage and subsequent cognitive impairment (Katsumoto et al., 2018; Tiwari et al., 2019). It is helpful for the diagnosis and treatment of AD to understand how related molecules participate in these processes. Due to the rapid development of high-throughput RNA-seq technologies, the dynamic expression patterns and functions of circRNAs in AD have been characterized recently in a large number of studies (Dube et al., 2019; Cervera-Carles et al., 2020; Cochran et al., 2021).

#### Differential expression of circRNAs in AD

circRNAs easily accumulate in the aging brain. Of the numerous changes in circRNAs, which ones are only related to aging and which ones are related to the development of AD needs to be clarified. Therefore, microarray or RNA-sequencing data from different brain regions of AD and age-matched healthy persons, including the frontotemporal lobar, entorhinal cortex, parietal cortex, posterior cingulate, hippocampus, and parahippocampal gyrus, were fully analyzed (Sekar et al., 2018; Dube et al., 2019; Cervera-Carles et al., 2020; Lo et al., 2020; Cochran et al., 2021; Urdanoz-Casado et al., 2021) (Table 1). Many AD-related circRNAs have been confirmed, and most of them are enriched in neurotrophic signaling, Aβ clearance, neuroinflammation, and oxidative stress. In addition to the brain parenchyma described above, abnormal changes in circRNAs in the cerebrospinal fluid and peripheral blood of AD patients have also been observed (Li et al., 2020a,b; Liu et al., 2020) (Table 1). Although it remains unknown whether the occurrence of AD is caused by the changed circRNAs in the brain, they may serve as biomarkers for the prediction or diagnosis of AD. Additionally, differential expression profiles of circRNAs in some AD cellular or animal models, including Amyloid precursor protein (APP)/PS1, Tg2576, 5xFAD, SAMP8, or Aβ administration, were constructed (Zhang et al., 2017, 2021; Wang et al., 2018; Lee et al., 2019; Ma et al., 2019; Nam et al., 2019) (Table 1). However, few dysregulated circRNAs have been found in common in both AD human and animal models according to these studies.

**TABLE 1:** Studies profiling circRNAs in AD.

Experimental subject	Sample	Method	Summary of dysregulated circRNAs (AD vs. control)	GO and KEGG enrichment function	References
28 AD patients and 16 matched healthy control.	Entorhinal cortex	RT-qPCR	circHOMER1 ↓	Aβ deposition	Urdanoz-Casado et al., 2021
83 AD patients and 13 matched healthy control.	Parietal cortex	RNA-seq	14 ↓ (*cirHOMER1) 19 ↑ (*circCDR1-AS)	Co-express with AD-relevant genes (APP and SNCA) and pathways (AD and oxidative phosphorylation)	Dube et al., 2019
4 male Tg2576 mice (7 M age) and 4 matched normal male C57BL/6 mice; 4 male Tg2576 mice (12 M age) and 4 matched normal male C57BL/6 mice.	Whole brain	Microarray	7 M (55 ↑ 46 ↓) 12 M(5 ↑ 7 ↓)	7 M (↑: immune activation, activation of inflammatory cascade, cellular adhesion, production of reactive oxygen species; ↓: progenitor self-renewal and neuronal differentiation and maintenance). 12 M (↑: immune activation, inflammatory response, cellular adhesion; ↓: synapse function and preservation of neuronal networks).	Lee et al., 2019
54 AD patients (19 sporadic AD, 9 autosomal dominant AD, 16 FTLD-TDP43, and 10 FTLD-Tau) and 15 control.	Frontotemporal lobar	RT-qPCR	circDOCK1 ↑ circHOMER1 and circKCNN2 ↓	/	Cervera-Carles et al., 2020
10 AD patients and 10 healthy control.	Posterior cingulate astrocyte	RNA-seq	No changed circRNAs	/	Sekar et al., 2018
8 AD patients and 8 matched healthy control.	Cerebrospinal fluid	Microarray	51 ↓ (* circ-PCCA, circ-HAUS4, circ-KIF18B, and circ-TTC39C). 112 ↑ (*circ-LPAR1, circ-AXL and circ-GPHN).	Neurotrophin signaling pathway, natural killer cell mediated cytotoxicity and cholinergic synapse.	Li et al., 2020a
5 AD patients and 5 matched healthy control.	PBMCs	Microarray	2070 ↓ (*hsa_circRNA_004884, 406961, 404524, 038632, 048474, 405836, 007215, 019252, 405383, and 101820). 1990 ↑ (*hsa_circRNA_402265, 404757, 101543, 004561, 092423, 406368, 003553, 402986, 404144, and 404225)	Inflammation, metabolism, and immune responses.	Li et al., 2020b
50 AD patients and 50 matched healthy control and 20 patients with dementia with Lewy bodies, and 40 patients with vascular dementia.	Peripheral blood	Microarray	10 ↓ (*hsa_circ_0003391, 0066336, and 0066331) 5 ↑	/	Liu et al., 2020
(1) 6 M APP/PS1 mice and matched wild-type control mice (*n* = 3 each group). (2) 9 M APP/PS1 mice and matched wild-type control mice (*n* = 3 each group).	Cerebral cortex	RNA-seq	6 M: 151 ↓ 192 ↑ 9 M: 102 ↓ 141 ↑	GO: synapse, cytoskeleton, postsynaptic density, cell–cell adherens junction, dendrite, axon, and neuron projection; KEGG pathways: cAMP signaling, MAPK signaling, insulin secretion, hippo signaling, adherens junction, focal adhesion, dopaminergic synapse, and PI3K-Akt signaling pathways.	Ma et al., 2019
7 M SAMP8 (AD model, *n* = 3) and SAMR1 (control, *n* = 3)	Cerebral cortex	RNA-seq	141 ↓ 94 ↑	GO: axon terminus, synapse, neuron projection terminus, axon part, and long-term synaptic depression.	Zhang et al., 2017
10 M SAMP8 (AD model, *n* = 3), 5 M SAMP8 (*n* = 3), and SAMR1 (control, *n* = 3).	Hippocampus	Microarray	10 M SAMP8 vs. control (40 ↓ *mmu_circRNA_017963, 45 ↑). 10 M SAMP8 vs. 5 M SAMP8 (121 ↓ 110 ↑)	GO: autophagosome assembly, exocytosis, apoptotic process, transport and RNA splicing. KEGG: synaptic vesicle cycle, spliceosome, glycosaminoglycan, Hepatitis B and SNARE inter- actions in vesicular transport.	Huang et al., 2018a
8 M APP/PS1 (*n* = 3) and the wild-type mice (*n* = 3)	Hippocampus	RNA-seq	26 ↓ 44 ↑	GO: most enriched in biological metabolic processes. KEGG: the cGMP-PKG signaling pathway, cAMP signaling pathway, axon guidance, platelet activation, LTP, hippo signaling pathway, and phosphatidylinositol signaling system. KEGG	Zhang et al., 2021
AD rats (intracerebroventricular injections of Aβ_1–42_ oligomers, *n* = 10) and control group rats (*n* = 10).	Hippocampus	Microarray	111 ↓ 444 ↑	GO: the extracellular region part, toll-like receptor binding, and the regulation of biological quality KEGG: AMPK and p53 signaling pathways.	Wang et al., 2018

*Represents the key circRNAs.

#### Role of circRNAs in AD

To date, the pathogenesis of AD is still unclear. The cascade of pathological reactions caused by amyloid aggregation is still the focus of AD research. Recently, the effects of circRNAs on AD pathology have mainly been explored in terms of Aβ production and clearance, neuroinflammation, and oxidative stress.

#### CircRNAs were associated with the production and clearance of Aβ in AD

Excessive deposition of Aβ in the cerebral cortex accelerates the development of AD (Alcendor, 2020). Aβ protein comes from the cleavage of APP by β-secretase and γ-secretase, the clearance of which is mainly dependent on the function of autophagy in neural cells (Baranello et al., 2015; Chen et al., 2017; Xin et al., 2018). GO enrichment analysis of some RNA sequencing data showed that many dysregulated circRNAs in AD were involved in the regulation of Aβ production and clearance. CiRS-7, a circRNA highly expressed in the human brain, has been proven to be downregulated in the brains of AD patients (Lu and Xu, 2016). Using HEK293T and SH-SY5Y cells, Shi et al. (2017) found that dysregulated ciRS-7 promoted the degradation of APP and beta-site amyloid precursor protein cleaving enzyme 1(BACE1) via the proteasome and lysosome in an NF-κB-dependent manner, which finally reduced the generation of Aβ. CircCwc27 could also be directly bound with purine-rich element-binding protein A, which suppressed the expression of a cluster of AD genes (Song et al., 2022). Interestingly, while studying the mechanism of Aβ production, a circRNA that can encode a polypeptide was found. This circRNA could be efficiently translated into a novel Aβ-containing Aβ175 polypeptide, which will be processed into Aβ (Mo et al., 2020). These results indicated an alternative pathway of Aβ production induced by a circRNA. In terms of Aβ clearance, the role of ciRS-7 was also referred to. It showed that ciRS-7 could sponge miR-7 and affect the expression of its target ubiquitin protein ligase UBE2A, a protein essential in the clearance of amyloid peptides in AD (Zhao et al., 2016). Moreover, circRNAs can change autophagy ability and indirectly regulate Aβ clearance and reduce the phosphorylation of Tau (Inoue et al., 2012; Lonskaya et al., 2014; Diling et al., 2019). For example, circRNA NF1-419 enhanced autophagy by binding dynamin-1 and adaptor protein 2 B1 in AD-like mice and reduced the expression of AD marker proteins, including Tau, p-Tau, Aβ, and APOE (Diling et al., 2019).

#### CircRNAs were associated with neuronal injury in AD

The loss of nerve cells is one of the typical features of AD, which is mainly caused by neuroinflammation and oxidative stress. Several studies have confirmed some roles of circRNAs in these processes. circ_0002945 was overexpressed in AD patient serum, Aβ-stimulated SK-N-SH cells and human primary neurons. Inhibition of circ_0002945 attenuated Aβ-induced cell apoptosis and endoplasmic reticulum stress through a novel circ_0002945/miR-431-5p/TNFAIP1 ceRNA network (Li G. et al., 2022). Similarly, using Aβ-treated SK-N-SH and SK-SY5Y cell lines, a study suggested that circRNA AXL increased neuronal injury and inflammation by targeting microRNA-328-mediated BACE1 in AD (Li Y. et al., 2022). The presence of Aβ and NFTs promotes microglial cell activation, leading to a persistent and robust release of inflammatory cytokines and chemokines and finally the death of surrounding neurons (Agrawal and Jha, 2020; Perea et al., 2020; Xu Y. et al., 2022). In a microglial cell line, BV2 cells, knockdown of circ_0005835 downregulated neuroinflammation by sponging miR-576-3p and changed the process of AD (Xu X. et al., 2022). In terms of oxidative stress, circLPAR1 and circ_0001588 have been proven to regulate oxidative stress in rat models and *in vitro* models of AD by sponging miRNAs (Zhu et al., 2020; Wu et al., 2021). In addition to the above indirect effects of circRNAs on neuronal morphology, circRNAs can directly regulate neuronal survival or neurite growth and branching in AD. CircTulp4 predominantly localizes in the nucleus, positively regulates hippocampal neurite growth and branching by changing the transcription of its parental gene, Tulp4, and might participate in the development of AD (Ma et al., 2021). It has been found that circ_0000950 decreased the expression of miR-103 and increased the expression of Ptgs 2 in AD patients, as well as promoted neuronal apoptosis, inhibited the growth of neuronal processes, and enhanced the level of inflammatory cytokines (Yang et al., 2019). The regulatory roles of circRNAs in neuroinflammation, oxidative stress, and neurite growth imply that circRNAs may function as important players in the pathogenesis of AD. Certainly, additional studies are still required to test the hypotheses in humans.

#### Future perspectives of circRNAs in AD

In view of the changes and biological roles of circRNAs in AD, as well as its high stability, it is expected to be used in clinical diagnosis and as a therapeutic target of drugs. Some studies have made useful attempts in this regard. Hsa_circ_0003391 was significantly downregulated in the peripheral blood of AD patients. Receiver operating characteristic curve analysis showed that hsa_circ_0003391 in peripheral blood or circ-AXL and circ-GPHN in cerebrospinal fluid had a relatively high area under the curve (AUC) value, suggesting a promising biomarker for AD diagnosis (Liu et al., 2020). Moreover, circPSEN1 was reported to be able to discriminate autosomal dominant AD from sporadic AD and controls with an AUC above 0.70. These results showed that circRNAs expression levels in body fluids are significantly correlated with both the occurrence and clinical severity of AD. Although there has been some progress in diagnosis, the evidence is still weak and needs to be further supported by large, multicenter studies (Chen et al., 2022). In addition, circRNAs, combined with other biomarkers and imaging tools to improve the diagnostic power, should also be considered.

Recently, direct intracerebral injection or *in vitro* cellular intervention and certain circRNAs have been confirmed to improve AD symptoms. In addition, the roles of circRNAs in AD drug treatment have also been reported, although disease-associated proteins are currently the main targets for AD treatment. Breviscapine, combined with bone marrow mesenchymal stem cell treatment, can reduce Aβ deposition and promote the degradation of APP and BAEC1 by regulating the circRNA ciRS-7 (Sun et al., 2023). PNS significantly inhibited the pathological progression of AD by upregulating five circRNAs or downregulating two circRNAs in AD mouse models (Huang et al., 2018b). Similarly, in human neuronal cells, berberine attenuated Aβ42-induced neuronal damage by regulating the circHDAC9/miR-142-5p axis (Zhang N. et al., 2020). Of course, the evidence from *in vitro* and animal experiments is still inadequate to prove the clinical efficacy of circRNAs as therapeutic drugs. In the future, more work should be done in AD patients, and targeting circRNAs may provide new ideas for the struggling development of AD drugs.

## Discussion

The recent discovery of circRNAs in the mammalian brain further deepens our understanding of the non-coding RNA regulatory system. Not only the spatiotemporal expression and distribution of circRNAs but also their various biological roles imply a close relationship between circRNAs and higher-order cognitive functions of the brain. However, until now, little direct evidence has indicated the role of circRNAs in cognitive abilities, such as reasoning, perception, memory, verbal and mathematical ability, and problem solving. In the future, using specific cognitive behavioral models, evaluating whether and how circRNAs are involved in these physiological functions under normal conditions is of great importance for clarifying the role of circRNAs in cognition. Given the closer kinship and similar complex cognitive functions between human and non-human primates, primates such as macaques are an ideal animal model for studying the above tissues.

Regarding the clinical application of circRNAs in cognitive-related diseases, although much progress has been made, there is still a long way to go. Increasing evidence indicates that circRNAs expression levels have potential value as non-invasive clinical biomarkers for central nervous system disorders. However, the evidence may not be solid. First, most of the studies were performed only in a single center with a relatively small number of cases. Second, the sensitivity and specificity of selected circRNA assemblies are not very good, so more specific circRNAs need to be screened and confirmed in multicenter, large-scale trials. Additionally, combining diagnosis with other clinical indicators, such as imaging indicators, should be considered to improve the accuracy of diagnosis. Several of the latest studies, using an exosome drug delivery system that directly regulates the expression of circRNA in the brain, achieved good efficacy in the progress of anti-depressant and anti-stroke interventions in mouse models, which may shed light on the future treatment of cognitive-related diseases with circRNAs by this convenient and efficient method.

## Author contributions

XY, HL, NC, WF, and ZG wrote the manuscript. YW conceived, coordinated the project, and revised the manuscript. All authors contributed to the article and approved the submitted version.
